# Kimura Disease: A Case Report and Review of the Literature with A New Management Protocol

**DOI:** 10.4061/2010/673908

**Published:** 2011-03-07

**Authors:** Mohamed Ashraf Fouda, Osama Gheith, Ayman Refaie, Mohamed El-Saeed, Adel Bakr, Ehab Wafa, Mona Abdelraheem, Mohamed Sobh

**Affiliations:** Mansoura Urology and Nephrology Center, Mansoura University, Mansoura, Egypt

## Abstract

Kimura disease (KD) is a chronic inflammatory disorder with angiolymphatic proliferation, usually affecting young men of Asian race but is rare in other races. The etiology of KD is still unknown. It is often accompanied by nephrotic syndrome. Herein, we present an atypical manifestation of Kimura disease occurring in a Caucasian man with steroid-responsive early membranous glomerulonephritis. Kimura disease can present atypically in a middle-aged Caucasian man with secondary steroid-responsive nephrotic syndrome. Steroid, endoxan, and MMF can be used safely and successfully in such situation. The diagnosis of KD can be difficult and misleading, and patients with this disease are often evaluated using avoidable procedures by just not being aware of KD.

## 1. Introduction

Kimura disease (KD) is a chronic inflammatory disorder with angiolymphatic proliferation, usually affecting young men of Asian race but is rare in western countries [1]. It is often accompanied by nephrotic syndrome and is a rare, chronic inflammatory disorder of unknown cause [2].

Chen et al. [3] reported 21 histopathology specimens conducted in the United States by the US Armed Forces Institute of Pathology and concluded that, though rare, if clinically suspected, KD should be included in the differential diagnosis for people of any racial group. KD is usually seen in young adults, with most patients being aged between 20 and 40 years of age; men are affected more commonly than women, with a 3 : 1 ratio [4].

The etiology of KD is still unknown but may be due to impairment or interference with immune regulation, atopic reaction to a persistent antigenic stimulus by arthropod bites, virus, and neoplasm. The most interesting hypothesis suggests Candida acting as a source of persistent antigenaemia, although neither hyphae nor spores have been isolated. The disease is manifested by hyperplasia of lymphoid follicles and vascular endothelium. Peripheral eosinophilia and the presence of eosinophils in the inflammatory infiltrate suggest that KD might be a kind of hypersensitivity reaction. With lymphocyte, T-helper 2 (Th2) might play a role [4].

Patients with KD may present with a solitary enlarged painless lymph node or generalized lymphadenopathy (67% to 100%) [2]. It classically presents as a nontender subcutaneous swelling in head and neck region, predominantly in preauricular and submandibular area. It may be associated with lymphadenopathy (both local and distal), marked peripheral eosinophilia, and an elevated IgE level [5].

Coexisting renal disease is common, with an incidence ranging from 10% to 60% [6], while 10% to 12% of patients may suffer from nephrotic syndrome [6] characterized by clinically relevant proteinuria in 12% to 16% of cases [7]. Renal impairment is probably due to immunocomplex-mediated damage or to Th2-dominant immune response disorders.

Herein, we present an atypical manifestation of Kimura disease occurring in a Caucasian man with steroid-responsive early membranous glomerulonephritis. 

## 2. Case

Fifty-year-old male Egyptian engineer who was suffering from hypertension since 10 years has recently presented to our outpatient clinic—in Mansoura Urology and Nephrology Center—with generalized anasarca, renal impairment after 18 months of the appearance of multiple small nontender masses at his forehead, temporal region, and behind the left ear. He gave no past history of chronic diarrhea suggesting inflammatory bowel disease.

On clinical examination, he was moderately obese (BMI: 35), and his blood pressure was controlled on his previous medications. Clinical examination revealed bilateral basal crepitations and moderate edema of both lower limbs.

His laboratory assessment showed that his renal function was impaired (creatinine, 2.4 mg/dL), normal electrolytes, low serum albumin (0.9 mg/dL), high cholesterol (280 mg/dL), proteinuria (8.9 grams/day). His hemoglobin was normal and also the total white cell count but his differential count showed eosinophilia (20%).

The virology profile was negative regarding hepatitis B and C, cytomegalovirus, and HIV. The immunological status revealed negative Antidouble strands DNA and both anticytoplasmic neutrophilic antibodies (ANCA-P and C). However, erythrocyte sedimentation rate was markedly elevated, tumor markers were normal (prostatic-specific antigen, carcinoembryonic antigens, and alpha fetoprotein), and IgE level was elevated.

Renal ultrasound evaluation revealed average sized kidneys with grade I echogenicity of the right kidney but normal left kidney.

**Figure 1:**
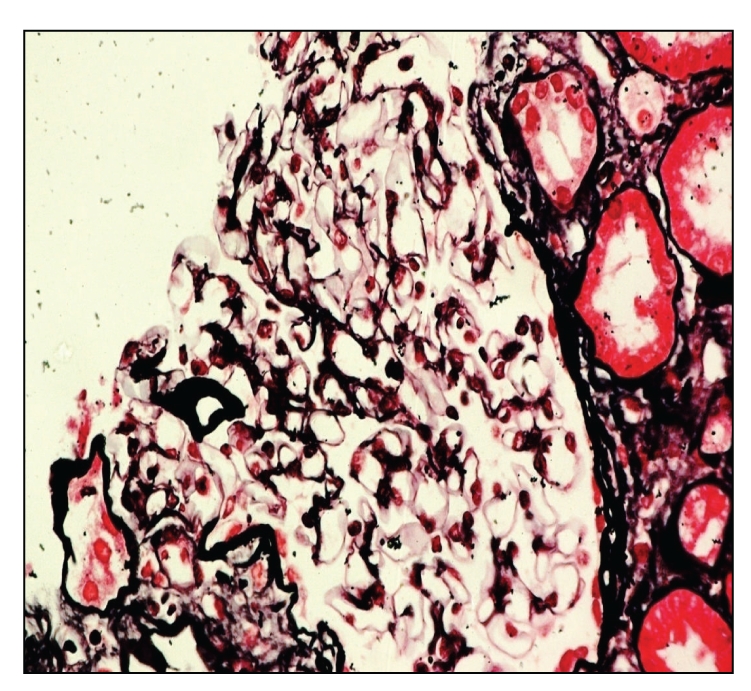
Irregular and thick basement membrane (H&E) and vaculizations of basement membrane of glomerulus (silver stain).

**Figure 2:**
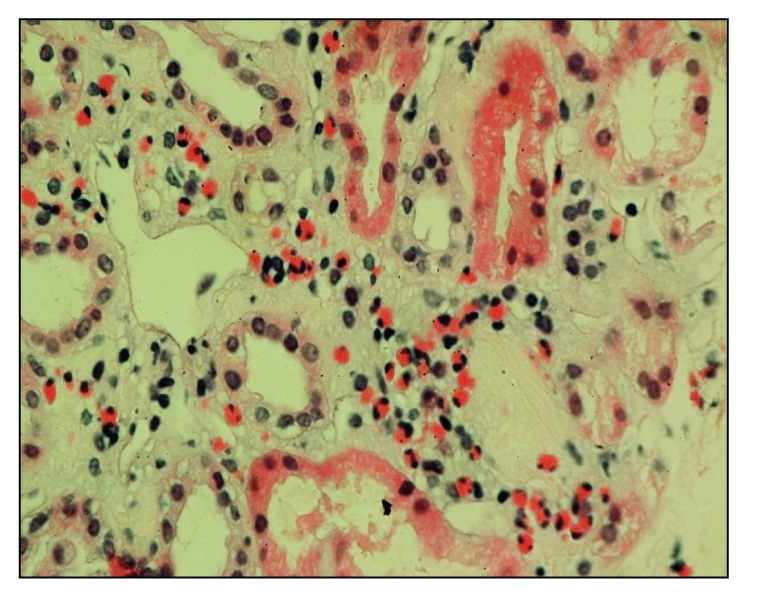
Eosinophilic interstitial infiltrates in renal tissue.

**Figure 3:**
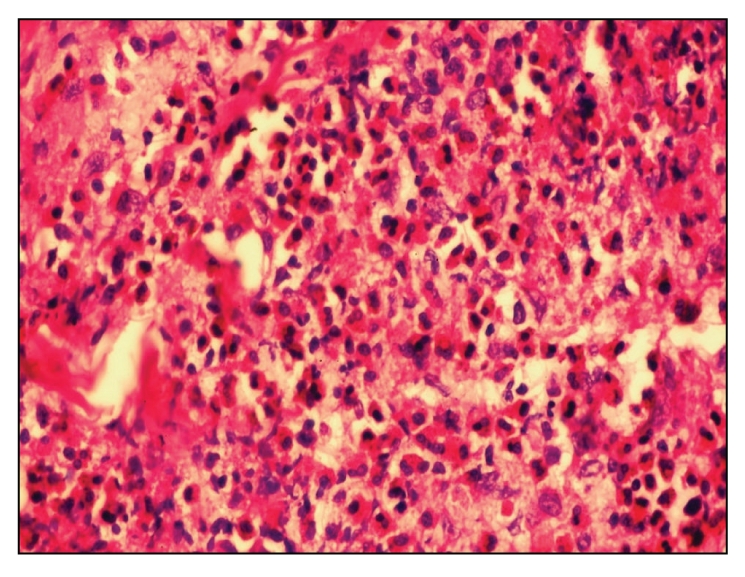
Eosinophilic infiltrates of skin lesion.

CT chest and MRI excluded evidence of underlying malignancy or lymphadenopathy. Therefore, ultrasound guided renal biopsy was carried out and light microscopic examination showed only 4 glomeruli/section. All of them were slightly enlarged but normocellular with occasional capillary loops showing small epithelial projections on silver methenamine stain, in addition to eosinophilic interstitial infiltrates (Figures 1 and 2), hence the diagnosis of early membranous nephropathy. Unfortunately immunofluorescence and electron microscopy could not be performed and the patient refused rebiopsy. Excisional biopsy from the skin lesions at the temporal region was revised and revealed eosinophilic interstitial infiltrates (Figure 3) with blood vessels in lymphoid follicles (angiolymphoid hyperplasia).

So the two biopsy findings were correlated and the final diagnosis was membranous nephropathy secondary to Kimura disease. The plan of management was intravenous cyclophosphamide, high dose steroids (60 mg/day) for 2 months, then gradual tapering, mycophenolate mofitel (2 grams/day), and histamine −1, Chlorpheneramine 12 mg; ranitidine 150 mg BD receptor blockers. The patient responded well to the previous anagement with complete disappearance of skin lesions and normalization of kidney function and disappearance of proteinuria (protein in urine less than 200 mg perday). Currently, the patient is on only a small dose of prednisone (7.5 mg/day). 

## 3. Discussion

Kimura disease (KD) is a rare chronic inflammatory disorder with angiolymphatic proliferation, usually affecting young men of Asian race and coexisting renal disease is common, with an incidence ranging from 10% to 60% [1, 6].

Still it is of unknown etiology; a number of theories have suggested impairment or interference with immune regulation, atopic reaction to a persistent antigenic stimulus by arthropod bites, virus [4], and neoplasm. The most interesting one is that Candida acts as a source of persistent antigenemia even subclinical [4].

Our case developed nephrotic syndrome with renal dysfunction after 18 months of onset of Kimura disease. Renal biopsy showed early membranous GN, in addition to eosinophilic interstitial infiltrates.

The patient was responsive to steroids, with disappearance of proteinuria 7 months after initiation of treatment. This was matched with what reported by the US Armed Forces Institute of Pathology [4] in most of the 20 other cases reported as the onset of nephrotic syndrome occurred after subcutaneous masses. Renal biopsy in 13 cases showed mesangial proliferative glomerulonephritis in 9, minimal change disease in 3, and membrane nephropathy in 2 cases. Serum creatinine levels were elevated only in 5 patients.

In our patient skin biopsy showed angiolymphoid hyperplasia with eosinophils which was matched with other reports of Kimura disease [8].

KD is often associated with autoimmune diseases such as ulcerative colitis and more frequently, unlike our case, bronchial asthma [6], typically responding to steroids [9]. In our case, we failed to diagnose the etiology after exclusion of autoimmune disease.

Coexisting renal disease is common, with an incidence ranging from 10% to 60% [7], while 10% to 12% of patients may suffer from nephrotic syndrome [6] characterized by clinically relevant proteinuria in 12% to 16% of cases [7]. Renal impairment is probably due to immunocomplex-mediated damage or to Th2-dominant immune response disorders.

The diagnosis of KD is not easy, and differential diagnosis includes inflammatory and neoplastic conditions, tuberculosis, cylindroma, dermatofibrosarcoma protuberans, Kaposi's sarcoma, pyogenic granuloma, and other infectious lymph node enlargements for example, toxoplasmosis.

Ultrasound, CT, and magnetic resonance imaging (MRI) might be diagnostic and can help staging the extent and progression of the disease as well as the lymph node involvement.

Because of the rarity of KD in Western countries, both clinicians and radiologists are relatively unfamiliar with some pathognomonic findings of this disease, thus leading to unnecessary diagnostic tests and investigations [7].

The diagnostic challenge of KD is generally solved by histological study: although there is no specific diagnostic feature of Kimura disease, fine-needle aspiration cytology is helpful in some cases, and definitive diagnosis can be obtained by histological examination of the excised lesion [4]. These findings were quite evident in all biopsies taken from our patient (skin and renal) which showed pronounced eosinophilic infiltrates, angiolymphoid hyperplasia eosinophilic renal interstitial infiltrates, and early membranous nephropathy, respectively [10]. 

Treatment for Kimura disease includes surgical resection and regional or systemic steroid therapy. Cytotoxic therapy and radiation have also been utilized. The disease has an excellent prognosis, although it may recur locally [11]. Sun et al. [12], reported that Imatinib—previously to be useful for treatment of hypereosinophilic syndrome and may work by selectively blocking protein-tyrosine kinases—might be an effective drug for the treatment of the disease. 

Our patient was subjected to nonradical surgical excision of the temporal skin lesion without recurrence especially after medical treatment. Treatment of KD is still controversial: surgical excision of nodules is the first-line treatment, with recurrence rate up to 25%; in any case, because of the lack of malignant transformation, radical or demolitive surgery should be avoided [4].

Systemically administered steroids show good effects on disease progression. Due to its effects on Th2 lymphocytes, cyclosporine has been described as a possible therapy for KD [13].

We tried single-pulse intravenous cyclophosphamide, honestly before correlating the skin and renal biopsies, then the patient was maintained on mycophenolate mofetil (MMF) and high dose systemic steroid with satisfactory response. This was not in agreement with that reported by wang et al. [2] that Kimura disease-associated nephrotic syndrome patients are sensitive to prednisone therapy but are likely to relapse and that in patients with recurrent nephrotic syndrome, renal insufficiency is not uncommon. This difference could be explained by genetic factors and different immunosuppressive protocol used in management.

This report is a singular manifestation of KD: our patient presented with atypical KD with renal affection that responded well to our immunosuppressive regimen without relapse. 

## 4. Conclusion

Kimura disease can present atypically in a middle-aged Caucasian man with secondary steroid-responsive nephrotic syndrome. Steroid, endoxan, and MMF can be used safely and successfully in such situation. The diagnosis of KD can be difficult and misleading, and patients with this disease are often evaluated using avoidable procedures by just not being aware of KD.
